# Antifungal Activity and Action Mode of Cuminic Acid from the Seeds of *Cuminum cyminum* L. against *Fusarium oxysporum* f. sp. *Niveum* (FON) Causing Fusarium Wilt on Watermelon

**DOI:** 10.3390/molecules22122053

**Published:** 2017-11-30

**Authors:** Yang Sun, Yong Wang, Li Rong Han, Xing Zhang, Jun Tao Feng

**Affiliations:** 1 Research and Development Center of Biorational Pesticide, Northwest A & F University, Yangling 712100, China; sunyang136592@nwsuaf.edu.cn (Y.S.); wy2010102163@163.com (Y.W.); hlr4119@126.com (L.R.H.); zhxing1952@gmail.com (X.Z.); 2 Engineering and Research Center of Biological Pesticide of Shaanxi Province, Yangling 712100, China

**Keywords:** watermelon fusarium wilt, *p*-isopropylbenzoic acid, biofungicide, disease management

## Abstract

In order to develop a novel biofungicide, the antifungal activity and action mode of cuminic acid from the seed of *Cuminum cyminum* L. against *Fusarium oxysporum* f. sp. *niveum* (FON) on watermelon was determined systematically. In this study, the median effective concentration (EC_50_) value for cuminic acid in inhibiting mycelial growth of FON was 22.53 μg/mL. After treatment with cuminic acid, the mycelial morphology was seriously influenced; cell membrane permeability and glycerol content were increased markedly, but pigment and mycotoxin (mainly fusaric acid) were significantly decreased. Synthesis genes of bikaverin (*Bike1*, *Bike2* and *Bike3*) and fusaric acid (*FUB1*, *FUB2*, *FUB3* and *FUB4*) both were downregulated compared with the control, as confirmed by quantitative RT-PCR. In greenhouse experiments, cuminic acid at all concentrations displayed significant bioactivities against FON. Importantly, significant enhancement of activities of SOD, POD, CAT and decrease of MDA content were observed after in vivo cuminic acid treatment on watermelon leaves. These indicated that cuminic acid not only showed high antifungal activity, but also could enhance the self-defense system of the host plant. Above all, cuminic acid showed the potential as a biofungicide to control FON.

## 1. Introduction

Watermelon is one of the most important fruits worldwide. In China, watermelon cultivation has been increasing year by year due to its comparatively high economic value and increasing consumption, but it is susceptible to fusarium wilt disease in continuous monocropping cultivation systems [1]. Watermelon fusarium wilt caused by *Fusarium oxysporum* f. sp. *niveum* (FON) is a destructive soil-borne disease leading to serious economic losses and limiting watermelon production throughout the world [2].

Importantly, FON is difficult to eliminate from soil. Laboratory studies has reported that three biological forms of *F. oxysporum* could survive morphologically unchanged for 11 or more years [3]. More than 50% of *Fusarium* species are toxigenic and produce harmful secondary metabolites (SM), such as the pigments fusarubins and bikaverin [3], as well as the mycotoxins, fumonisins, fusarins [4], and fusaric acid [5,6]. In the progression of the infection, fusarium species damage host plants through intrusion of hyphae into host vascular system, secretion of hydrolytic enzymes and mycotoxins which lead to watermelon root and stem necrosis, cellular apoptosis, foliar wilting and then death within a few weeks [7,8].

Due to the fact FON can survive for several years in soil as chlamydospores and many hosts are symptomless [9], fusarium wilt is difficult to control, although traditional crop rotations are an effective strategy to control FON [10]. For many other pathogens, the application of fungicide has been a common and successful method for disease management, however, the application of fungicides should be phased out because of the increasing attention to environmental and human health and the development of fungicide resistance [9,11]. Some experiments have documented that fungicides have drastic effects on the soil biota and most cause a decline in soil fertility [12]. Consequently, alternative control strategies for this disease would be useful and urgent in reducing health hazards, environmental damage and the pollution potential [13]. Biofungicides may be an attractive alternative method for controlling this disease.

Biofungicides are living organisms (plants, microscopic animals such as nematodes, and microorganisms, including bacteria, fungi and viruses) or natural products derived from these organisms, that are used to suppress pest populations and pathogens [14]. Firstly, many studies have reported that using nonpathogenic *Fusarium* spp. fusarium wilts could be controlled [15]. Secondly, some antagonistic strains showed high bioactivities against fusarium wilt, such as *Trichoderma* spp. [16], *Bacillus* spp. [17] and *Aspergillus* spp. [18]. Thirdly, plant extracts or phytochemicals, such as essential oils, steroids, phenolic acids and alkaloids had good antifungal activities. For example, it has been reported that essential oils from pepper, cassia tree, mustard and clove could suppress disease development caused by *F. oxysporum* f. sp. *melonis* on muskmelon and reduce the population density of pathogen in greenhouse experiments [19]. Wu et al. found many benzoic acid analogues such as gallic acid, ferulic acid and *p*-hydroxybenzoic acid strongly inhibited FON growth [20,21,22].

Cuminic acid (*p*-isopropylbenzoic acid), isolated from the seed of *Cuminum cyminum*. L. [23], belongs to the benzoic acid chemical group [24]. In a previous study, it has been reported that cuminic acid possessed good inhibition towards several plant pathogens, such as *Sclerotinia sclerotiorum*, *Phytophthora capsici*, *Rhizoctonia cerealis*, and *Fusarium oxysporum*. EC_50_ values of cuminic acid against mycelial growth of *P. capsic* and *S. sclerotiorum* were only 19.7 µg/mL and 7.3 µg/mL, respectively [25], which were lower than the EC_50_ value of other benzoic acid derivatives in previous reports [20,21,22]. In pot experiments, after the application of cuminic acid at 1000 μg/mL, control efficacies of over 60% against *P. capsic* and *S. sclerotiorum* were obtained, which was comparable with the efficacy of metalaxyl (250 µg/mL) [24] and procymidone (100 μg/mL) [25].

Considering the broad spectrum and significant antifungal activity of cuminic acid and the difficult management of fusarium wilt, it’s necessary to evaluate cuminic acid as a potential biopesticide to control fusarium wilt on watermelon. The objectives of this research were to: (a) determine the effect of cuminic acid on FON colony growth; (b) evaluate the effect of cuminic acid on the morphological and physiological characteristics of FON; (c) test the efficacy of cuminic acid against FON in watermelon plant in greenhouse experiments, and study the effect of cuminic acid on the antioxidant defensive enzymes in watermelon plant subjected to fusarium wilt; (d) examine the effect of cuminic acid on differences in the transcription levels for FON genes associated with the biosynthesis of fusaric acid and bikaverin by a quantitative RT-PCR method.

## 2. Results

### 2.1. Effect of Cuminic Acid on FON Colony Growth

The effects of various concentrations of cuminic acid on the mycelial growth of FON are shown in Table 1, and cuminic acid were found to inhibit the mycelial growth of cuminic acid in a dose-dependent manner. Mycelial growth of FON was strongly inhibited by cuminic acid at a relative low concentration of 25 μg/mL. Based on log-transformation analysis, EC_30,_ EC_50_ and EC_70_ values were calculated as 5.6, 22.53 and 91.3 μg/mL, respectively.

### 2.2. Effect of Cuminic Acid on Mycelial Morphology of FON

**Figure 1:**
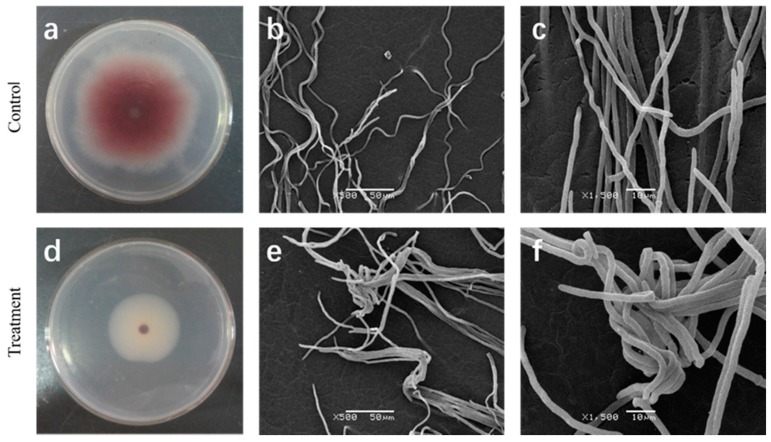
Effect of cuminic acid on mycelia morphology of FON. (**d**–**f**) Untreated plates; (**a**–**c**) Plates treated with cuminic acid at EC_50_ value (22.53 μg/mL). Values are means and standard errors.

A clear effect of the cuminic acid on mycelia morphology of FON was observed (Figure 1). After 7 days’ incubation, treatment with cuminic acid at the EC_50_, the color of mycelia was visible lighter than control (Figure 1a,d) in PDA plates, while the mycelia of the control were natural, uniseriate and uniform (Figure 1b,c) by SEM. For strains amended with cuminic acid at the EC_50_, mycelia were severely deformed, twining and clustered (Figure 1e,f).

### 2.3. Effect of Cuminic Acid on Cell Membrane Permeability of FON

**Figure 2:**
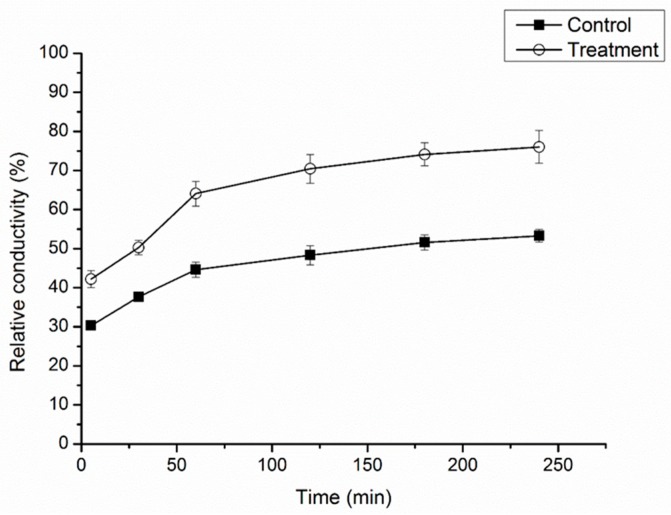
Mycelial relative conductivity of FON with or without cuminic acid treatment at the concentration of EC_50_ value (22.53 μg/mL). Values are means and standard errors.

To confirm the membrane-disruption effects of cuminic acid on the hyphal cells, the relative conductivity of the mycelia treated with cuminic acid were determined. As shown Figure 2, the relative conductivity of the mycelia treated with cuminic acid increased gradually during incubation, being about 45.78% higher than that of control after 120 min incubation.

### 2.4. Glycerol Content of Mycelia

**Figure 3:**
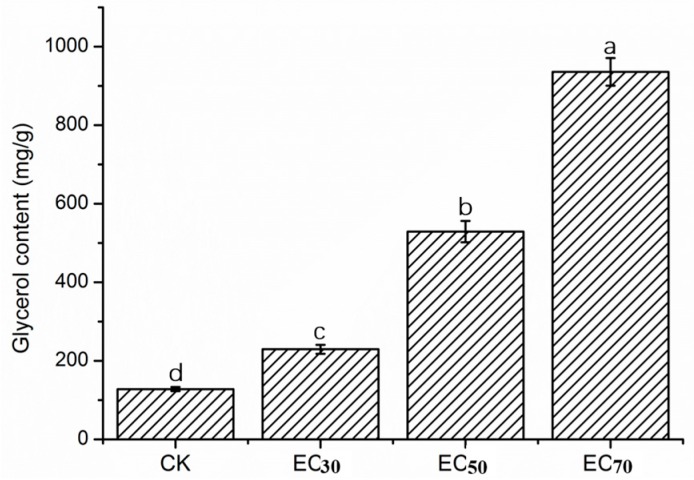
Glycerol content of mycelia of FON with or without cuminic acid treatment at concentrations of EC_30_ (5.6 μg/mL), EC_50_ (22.53 μg/mL) and EC_70_ (91.3 μg/mL). Bars denote the stand error of three experiments. Data represents means of three replicates with standard deviation. Data (means ± SD, n = 3) followed by the same letters in the row show no significant differences (small letters, *p* < 0.05).

After treatment with cuminic acid, the content of glycerol was always significantly higher than the control without cuminic acid treatment (Figure 3). As the concentration was increased, the glycerol content of the mycelia increased over time. The glycerol contents for three concentrations of cuminic acid (EC_30_, EC_50_ and EC_70_) significantly increased by 79.3%, 313.56% and 631.57%, respectively

### 2.5. Mycotoxin Concentration of FON in Liquid Culture

**Figure 4:**
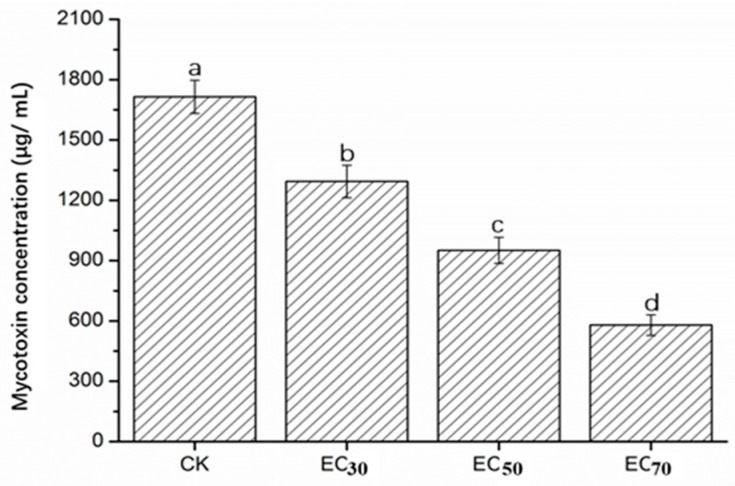
Mycotoxin production (mainly fusaric acid) concentration in FON with cuminic acid treatments at concentrations of EC_30_ (5.6 μg/mL), EC_50_ (22.53 μg/mL) and EC_70_ (91.3 μg/mL) in liquid culture. Bars denote the stand error of three experiments. Data represents means of three replicates with standard deviation. Data (means ± SD, n = 3) followed by the same letters in the row show no significant differences (small letters, *p* < 0.05).

Mycotoxin (mainly fusaric acid) concentration of FON in PDB was suppressed by cuminic acid treatment in a concentration dependent manner. Significant suppression was found even at the lower EC_50_ value concentration. The mycotoxin concentration was decreased by 24.57–66.22% compared with control (Figure 4).

### 2.6. Greenhouse Experiments

The effect of cuminic acid on FON was evaluated under greenhouse conditions (Table 2). Our experiments demonstrated that cuminic acid at all concentrations has a significant suppression effect on FON. In plants under cuminic acid at 2000 μg/mL we obtained a 21.5% disease index and 74.5% efficacy, which was not significantly difference from carbendazim at 1000 μg/mL. However, in plants under cuminic acid at 1000 μg/mL, the disease index and efficacy were 38.8% and 54.5%, which was lower than under carbendazim at 1000 μg/mL.

### 2.7. Assay of Defense Enzyme Activities and Malondialdehyde (MDA) Content

**Figure 5:**
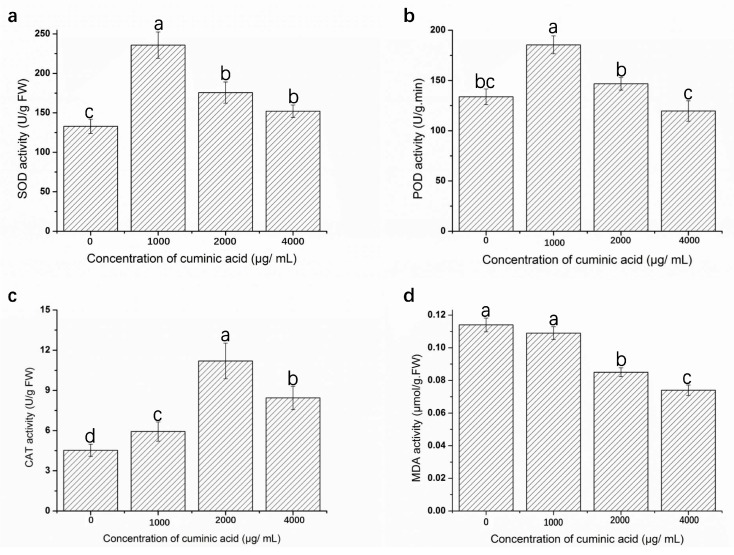
SOD (**a**); POD (**b**) and CAT (**c**) activities and MDA activity (**d**) of the watermelon leaves treated with cuminic acid at 0, 1000, 2000 and 3000 μg/mL, respectively. Data represents means of three replications with standard deviation. Data (means ± SD, n = 3) followed by the same letters in the row show no significant differences (small letters, *p* < 0.05).

The activities of superoxide dismutase (SOD), peroxidase (POD) and catalase (CAT) are shown in Figure 5a–c and the content of MDA is shown in Figure 5d. Activities of SOD, POD, CAT under cuminic acid treatment in watermelon leaves were enhanced in comparison with control, except for cuminic acid treatment at 4000 μg/mL in POD activities. SOD and POD activities experienced the trend in all the plants, and the highest enzyme activity (43.65%) was found in treatment with cuminic acid at 1000 μg/mL (Figure 5a) and a 27.87% increase was observed compared with control (Figure 5b).

As for CAT activity, the highest enzyme activity was found after cuminic acid treatment at 2000 μg/mL, which corresponded to a 59.55% (Figure 5c) increase compared to control. However, MDA content decreased steadily in all the samples during the whole experimental period with the increased concentration of cuminic acid.

### 2.8. Quantitative RT-PCR

**Figure 6:**
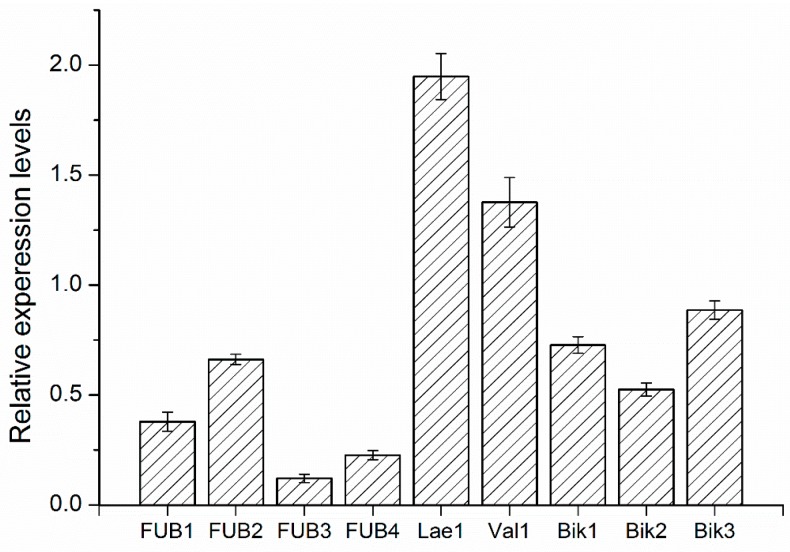
Gene expression level of synthesis of fusaric acid (*FUB1*, *FUB2*, *FUB3* and *FUB4*) and bikaverin (*Bike1*, *Bike2* and *Bike3*), and components of a velvet-like complex *(Lae1* and *Vel1*) relative to without treatment cuminic acid. Values are the means ± standard error (SE) of three repeated experiments.

To confirm whether the biosynthesis of fusaric acid and pigment in FON would be affected by cuminic acid, expression of genes including the ones involved in the synthesis of bikaverin (*Bike1*, *Bike2* and *Bike3*), fusaric acid (*FUB1*, *FUB2*, *FUB3* and *FUB4*) and components of a velvet-like complex (*Lae1* and *Vel1*) were quantified (Table 3). Relative to expression in the wild-type strain (Figure 6), synthesis genes of bikaverin (*Bike1*, *Bike2* and *Bike3*) and fusaric acid (*FUB1*, *FUB2*, *FUB3* and *FUB4*) both exhibited decreased expression compared with the internal control. 

Expressions of *FUB3*, *FUB4* and *Bike2* were about 0.88, 0.77 and 0.46 fold lower relative to the internal control. However, genes of components of a velvet-like complex (*Lae1* and *Vel1*) exhibited significantly increased expression (1.95 and 1.37-fold).

## 3. Discussion

In previous study, cuminic acid and cuminic aldehyde, as the major bioactive constituents of *C. cyminum* seed, were reported to possess broad spectrum antifungal activities [23,25]. Cuminic acid as a representative chemical of the benzoic acid group, also exhibited a significant antifungal activity and enhanced the defense capacity of plants against *Phytophthora capsic* [24]. This study was focused on the biochemistry and physiology alterations in *Fusarium oxysporum* f. sp. *niveum* mediated by cumunic acid, and confirms that this chemical has a value for development and utilization as a potential biofungicide.

In the current study, results showed that the growth of FON was strongly inhibited by cuminic acid in a concentration-dependent manner, with an EC_50_ value of 22.53 μg/mL. Cuminic acid exhibited a significant higher antifungal activity in PDA plates compared with other chemicals of the benzoic acid group, such as cinnamic acid [26], gallic acid [20] and sinapic acid [8]. Interestingly, we found that the color of mycelia in the strains treated with cuminic acid at the EC_50_ in PDA plates was visibly lighter than that in control, and mycelia were abnormal by SEM. In addition, the cell membrane permeability and glycerol content were significantly enhanced, which was consistent with the activity of cuminic acid against *Phytophthora capsic* [24], which indicated that the mechanism of action of cuminic acid against plant pathogens might be through damaging the mycelial structure and inducing intracellular plasma leakage.

Mycotoxin (mainly fusaric acid) production is widely distributed among the whole *Fusarium* species [27], particularly pathogenic strains of *F. oxysporum*. It is an important pathogenic factor causing wilt diseases in various plants, such as watermelon, tomato and cucumber. Importantly, there is an increased virulence to the host with the increase of mycotoxin production by *F. oxysporum* [28]. In the initiation of infection and symptom development, the toxins produced by pathogens were a pathogenicity determinant in FON [29]. In the current study, a significant reduction of mycotoxin was observed after treatment with cuminic acid, indicating that cuminic acid could reduce the pathogenicity of FON by inhibiting the secretion of mycotoxins (mainly fusaric acid). According to the reduction of pigments and fusaric acid production, we selected nine genes associated with the biosynthesis of fusaric acid [5,30] and pigments [3] to determine whether FON treatment with cuminic acid would affect the biosynthesis of fusaric acid and pigment by quantitative RT-PCR. Synthesis genes of bikaverin (*Bike1*, *Bike2* and *Bike3*) and fusaric acid (*FUB1*, *FUB2*, *FUB3* and *FUB4*) were both downregulated compared with the control, which was consistent with previous results. Previous studies have documented that genes of components of a velvet-like complex (*Lae1* and *Vel1*) participated in the biosynthesis of and modulate the expression of fusaric acid [3,5]. However, these significantly overexpressed genes in this study still need to be further studied.

In greenhouse experimenta, cuminic acid at all concentrations had a significant suppressive effect on FON. After treatment with cuminic acid at 2000 μg/mL, the disease index and efficacy were not significantly different from those after treatment with carbendazim at 1000 μg/mL, indicating that cuminic acid has significant antifungal activities against FON and possesses potential as a biofungicide.

Reactive Oxygen Species (ROS) are harmful to several cellular components and they can cause lipid peroxidation and induce membrane injury, then resulting in cell senescence [31]. Moreover, antioxidant enzymes such as SOD, POD, CAT play crucial roles in suppressing oxidative stress. When ROS increases, SOD directly catalyzes the conversion of O^2−^ into H_2_O_2_, which is then converted into water and oxygen by CAT [32], while POD decomposes H_2_O_2_ into H_2_O and O_2_. Meanwhile, the enzyme POD participates in the construction, rigidification and lignification of cell walls, which protects plant tissues from damage [33]. In addition, the high MDA reflects the higher production of H_2_O_2_ and ROS [34]. In the present study, activities of SOD, POD, CAT in watermelon leaves under cuminic acid treatment were significantly enhanced in comparison with control. Correspondingly, a decreased MDA content in cuminic acid treated was observed. These data clearly suggested that cuminic acid could prevent FON development and reduce the level of lipid peroxidation through a mechanism involving activation of antioxidant defensive enzymes.

In conclusion, cuminic acid has a high inhibition effect in the mycelial growth of FON on watermelon plants. Although further work is needed to entirely understand the mode of action of cuminic acid against FON, we can conclude that cuminic acid used in this study could be developed as a promising biofungicide.

## 4. Materials and Methods

### 4.1. Pathogen Strains and Fungicides

*Fusarium oxysporum* f. sp. *niveum* were collected from infected watermelon plant and maintained on potato dextrose agar (PDA) [24] medium provided by the Laboratory of Research and Development Center of Biorational Pesticide, Northwest A & F University. Cuminic acid (98%) and carbendazim (98.0%) used in the experiment were purchased from the Sigma Co. (St. Louis, MO, USA).

### 4.2. Effect of Cuminic Acid on FON Colony Growth

The effect of cuminic acid on colony growth was determined as follows: PDA media was amended with a series of cuminic acid addtions at the final concentrations of 0, 3.125, 6.25, 12.5, 25, 50 and 100 μg/mL. A 5-mm mycelial plug taken from the leading edge of 7-day-old colonies was inoculated into the center of the amended PDA medium. Plate was incubated in a growth chamber at 28 °C for 7 days, colony diameter was determined by measuring the average of two perpendicular directions on each plate. According to previous studies [24], the EC_50_ values were calculated by regressing percentage growth inhibition against the log of cuminic acid concentration. Each concentration was tested thrice with three replicates.

### 4.3. Effect of Cuminic Acid on Mycelial Morphology of FON

Mycelia plugs cut from the margin of 7-day-old colony were placed on PDA plates containing cuminic acid at the EC_50_ (22.5 μg/mL) for inhibition of mycelial growth. Control was plates without cuminic acid. After 7 days at 28 °C, the margin of medium area (10 mm × 10 mm) was placed on slide glass. High-resolution images of mycelial morphology changes in cuminic acid treated samples were obtained by scanning electron microscope (SEM, JSM-6360LV, JEOL, Tokyo, Japan) [35]. Three replicates were processed and the experiment was repeated twice.

### 4.4. Effect of Cuminic Acid on Cell Membrane Permeability of FON

Mycelial cell membrane permeability was expressed as the relative conductivity. Ten mycelial plugs were added into 250-mL flasks containing 100 mL of potato dextrose broth (PDB). The flasks were shaken at 180 rpm and 28 °C for 5 days, partial flasks were amended with cuminic acid at the EC_50_ (22.5 μg/mL). Control was flasks without cuminic acid. The flasks were shaken for 2 days, mycelia samples (0.5 g of mycelia) were collected by filtration through filter paper, and suspended in 20 mL of distilled water. Conductivity of the treated water was measured after 5, 30, 60, 120, 180, 240 min with a conductivity meter (CON510 Eutech/Oakton, Bukit Batok, Singapore), after 240 min, final conductivity was determined by mycelia were boiled for 5 min to completely kill the tissues and release all electrolytes and cooled to 25 °C. The experiment with three replicates was repeated three times. The relative conductivity was calculated as following formula [36]:

(1) $$\mathrm{Relative}\;\mathrm{conductivity}=\frac{\mathrm{Conductivity}\;\mathrm{at}\;\mathrm{different}\;\mathrm{times}}{\mathrm{Final}\;\mathrm{conductivity}\;}\times 100\%\;$$

### 4.5. Glycerol Content of Mycelia

Glycerol content was determined using the described method [37] with minor modifications. A standard curve for glycerol was obtained according to the described method. The mycelia of FON strain was prepared as described above. In addition, partial flasks were amended with cuminic acid at the EC_30_ (5.6 μg/mL), EC_50_ (22.5 μg/mL) and EC_70_ (91.3 μg/mL). Mycelia (0.5 g per sample) were ground with a frozen pestle and a mortar. The sample was washed thrice with autoclaved distilled water and transferred to 10-mL centrifuge tubes. The volume for each sample was adjusted to 10 mL with water. According to the standard curve, glycerol content of the sample was calculated. Each treatment was processed with three replicates, and the test was repeated three times.

### 4.6. Mycotoxin Conerntration of Mycelia

Mycotoxin (mainly fusaric acid) concentration was determined as described by Wu et al. [26]. A standard curve was prepared with standard of fusaric acid (Sigma Co.). Ten mycelial plugs were added into 250-mL flasks containing 100 mL PDB. The flasks were shaken at 150 rpm and 28 °C for 10 days, partial flasks were amended with cuminic acid at the EC_30_, EC_50_ and EC_70_, Control was flasks without cuminic acid. The flasks were continued to shake for 4 days, and the culture filtrate was collected after filtration. The culture filtrate was acidified to pH 2 with 2 M HCL and an equal volume of ethyl acetate was added, followed by vigorous shaking for 1 min, and held for 30 min. The collected organic phase was then placed in a new tube. The above procedure was repeated three times. The organic phase was centrifuged at 4000 rpm for 15 min and the supernatant was collected and dried. The dried residue was redissolved with ethyl acetate to 5 mL. By UV spectrophotometry (UV-5100 spectrophotometer, Yuan Xi, Shanghai, China), the OD_268_ was measured. Each treatment was processed with three replicates, and the test was repeated three times.

### 4.7. Preparation of FON Inoculum and the Watermelon Seedlings

Ten mycelial plugs were added into 250-mL flasks containing 100 mL PDB. The flasks were shaken at 150 rpm and 28 °C for 7–10 days, depending on experiments. The spore suspensions were filtered and adjusted to 1 × 106 cfu/mL with a hemacytometer (Thermal Fisher Scientific, Hennigsdorf, Germany).

The watermelon seeds were surface disinfected in sodium hypochlorite (5%, *w*/*v*) for 5 min, washed twice with sterile water and then germinated in a 9 cm diameter sterile plates containing wet filter paper. The germinated seeds were sown into each nursery cups (4 cm diameter, 6 cm high) containing a sterilized mixture of nursery soil, organic manure and sand (2:1:1, *w/w*). The seedlings were grown in greenhouse (natural light at 32/18 °C (day/night) and 50–70% humidity with). Seeding were watered when needed. Watermelon seedlings (two cotyledon period stage) were transplanted into pots (10 cm diameter, 15 cm high) containing enough sterilized mixture of nursery soil. The seedings (two true leaves stage) were used for all experiments.

### 4.8. Greenhouse Experiments

Experiments were completely randomized designs with five treatments. The five treatments were as follows: water, cuminic acid at 1000, 2000 and 4000 μg/mL, and carbendazim at 1000 μg/mL. 10 mL of treatment were poured into the plant root when 10 mL of FON spore suspension (10^6^ cfu/mL) was inoculated. During the procedure of treatment, plant roots were injured by minor vulnerable cuts. After 3 weeks of treatment, 10 watermelon plants per treatment were examined and disease severity was measured according to Rojan et al. [38]. The disease index and efficacy were calculated according to Zhao et al. [39]. Ten plants per treatment were applied and the experiment was repeated twice.

### 4.9. Assay of Defense Enzymes Activities and Malondialdehyde (MDA) Content

Watermelon leaves cut from the plants treated with cuminic acid in the above section were collected on ice. Three g of leaf per sample were homogenized and suspended in 8 mL of 0.5 mM phosphate buffer, pH 7.8, containing 0.2 mM EDTA and 2% PVPP and centrifuged at 100,000 rpm for 20 at 4 °C and the resulting supernatants were directly used for assay. POD and SOD activities were determined by the methods of Garcia-Limones et al. [40]. CAT activity was assyed following the procedures described by Sun et al. [41]. As for MAD content, the assay mixture consisted of 5% trichloroacetic acid (TCA) and 0.6% thiobarbituric acid (TBA). MDA concentration was determined according to the methods described by Heath and Packer [2]. Five leaves per treatment were used and the experiment was conducted twice.

### 4.10. Quantitative RT-PCR

Quantitative RT-PCR was carried out in FON to examine differences in transcript levels for genes associated with the biosynthesis of fusaric acid [5,30] and pigment [3]. The mycelia of FON strain was prepared as described in 2.4. Total RNA was isolated from mycelia of FON strain using a RNA extraction kit (Takara, Dalian, China) according to the manufacturer’s protocol. First-strand cDNA was generated from RNA using the Prime Script RT reagent kit (Takara). In this study, actin gene was set as the internal control, and all applied primers for qRT-PCR were listed in Table 3. qRT-PCR was carried out in a 20 μL reaction mixtures containing 12 μL SYBR Premix Ex Taq II (Takara), 0.8 μL of each primer and 1.6 μL templated DNA. All quantitative RT-PCRs were performed with an CFX96TM real-time detection system (Bio-Rad, Hercules, CA, USA). Each sample was run twice in three independent biological experiments. With a related actin gene (Foxq13729) as the reference gene, relative expression levels of target genes were calculated according to the 2^−^^△△Ct^ method [42].

### 4.11. Statistical Analysis

In this study, data from repeated experiments were combined for analysis, owning to the fact the variances between experiments were homogeneous. All data were processed and analyzed using SPSS 14.0 (SPSS Inc., Chicago, IL, USA) according to previous studies [24]. When ANOVAs were significant (α = 0.05), means were separated with Fisher’s least significant difference (LSD).

## Figures and Tables

**Table 1:** The effect of cuminic acid on FON colony growth.

Compounds	Regression Equation	EC_50_ (μg·mL^−1^)	Confidence Interval of EC_50_ (*p* < 0.05)	χ2
Cuminic acid	Y = 3.83 + 0.86X	22.53	17.85–25.96	4.83

Note: Data represents the mean value of triplication. The EC_50_ was assessed based on log-transformation analysis. Y: Probit-inhibition (%); X: log-dose.

**Table 2:** Effect of cuminic acid on control of fusarium wilt on watermelon.

Compound	Concentration	Disease Index (%)	Efficacy (%)
Cuminic acid	1000 μg/mL	38.8 ± 2.5b	54.5 ± 2.3b
	2000 μg/mL	21.4 ± 1.51c	74.5 ± 1.5a
	3000 μg/mL	24.8 ± 1.15c	71.9 ± 1.22a
Carbendazim	1000 μg/mL	23.2 ± 1.18c	72.8 ± 1.4a
Water	___	85.5 ± 3.5a	___

Note: Results are the means of 10 watermelon plants and from two independent experiments. Means followed by the same letters were not significant different according to LSD (*α* = 0.05).

**Table 3:** qRT-PCR primers applied in this study.

Gene Name	Accession Number	Primer	Sequence (5′-3′)
*Bike1*	AJ278141	Forward	CGGTATCTGTGGTGGTGTC
		Reverse	TCGGGAGGTGATGTTGTG
*Bike2*	AM229668	Forward	TGCCTGCTCCACAGTCTACG
		Reverse	GCCAATCTTGACCGCCAC
*Bike3*	AM229667	Forward	CGCCAAAGTCATCAAGGA
		Reverse	AGGCTCAGGCACCACAAA
*FUB1*	FFUJ_02105	Forward	ACTTCGCCTCGTCATCTC
		Reverse	GAACCCAGCATCAAACTTAT
*FUB4*	FFUJ_02108	Forward	CACCCTTGCTCATCACAG
		Reverse	CGTAAAAATATCCTTCCGAATAATC
*FUB2*	FFUJ_02106	Forward	GCCAACTGCTGTCACTAT
		Reverse	TTCCGAGGTGGAGATTAG
*FUB3*	FFUJ_02107	Forward	CCCGATACACCATACCCT
		Reverse	CCAACTTCTTGCCGTGAG
*Lae1*	FVEG_00539	Forward	TATTGGTACGGGCACAGG
		Reverse	GGCATAAAGCCAGGAGGA
*Vel1*	FN548142	Forward	CTACTAAGGAGGAAAGGGACT
		Reverse	TCCATCAAACCAGGAAACT
Related actin gene	Foxq13729	Forward	GAGGGACCGCTCTCGTCGT
		Reverse	GGAGATCCAGACTGCCGCTCAG
